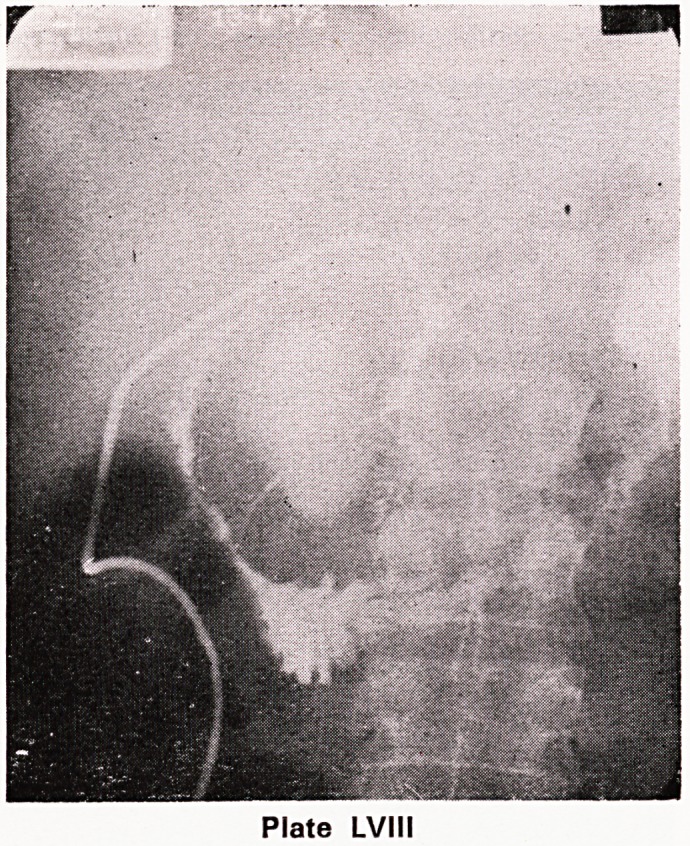# Solitary Cyst of the Pancreas and 'Reversible Diabetes Mellitus'

**Published:** 1974-10

**Authors:** Alban A. J. Barros D'Sa

**Affiliations:** Surgical Registrar, Musgrove Park Hospital, Taunton


### Solitary Cyst of the Pancreas and 'Reversible Diabetes Mellitus'

**Alban A. J. Barros D'Sa, M.B., Ch.B., M.R.C.S., L.R.C.P., F.R.C.S., Ed.,**
Surgical Registrar, Musgrove Park Hospital, Taunton*

**Introduction**

Although diabetes mellitus is a well recognised complication of chronic relapsing pancreatitis, it is uncommon in patients with pancreatic cysts, especially if these are solitary. In the rare instances where insulin or one of the oral anti-diabetic drugs is required, this is needed for life.

**Case Report**

A male aged 38 years was first seen in March 1971 with a year's history of intermittent attacks of epigastric pain. The pain worked round to both loins and was more or less constant for two or three days. He then had a few days freedom before the next attack. The pain was not affected by food or hunger but he had noticed that his bowels were not opened during the attacks, although between attacks they were opened twice daily. There were no urinary symptoms. He smoked 20 cigarettes a day and drank 7 pints of beer daily.

Clinically he was 15 stone in weight with evident pigmentation. His body hair was sparse, there was clubbing of the fingers and bilateral Dupuytren's contractures were present. Liver function tests showed normal bilirubin and alkaline phosphatase levels but a raised lactic dehydrogenase (L.D.H.) at 850 units/ml. His serum protein pattern showed a much elevated gamma globulin at 1.76 g/100 ml and subsequent liver biopsy confirmed an alcoholic type of cirrhosis.

His urine showed glycosuria and a glucose tolerance test showed mild diabetes. He was advised to restrict his carbohydrate intake and to stop drinking alcohol completely.

A cholecystogram was normal, a barium meal showed a healed duodenal ulcer but no other abnormality and a barium enema was normal.

The patient's glycosuria was controlled by a strict diet until early 1972. At this time his attacks of abdominal pain became more intense and his glycosuria more persistent in spite of carbohydrate restriction. He was started on treatment with Tolbutamide.

Two weeks after this he was admitted as an emergency with severe attacks of epigastric pain radiating to both loins, nausea and weight loss. He was jaundiced and his liver function tests showed an obstructive picture. His blood and urine showed very high sugar levels. Serum amylase was normal. His jaundice progressed over the next two weeks until his serum bilirubin was 5.7 mg/100 ml and his alkaline phosphatase 71 units. An epigastric mass was now palpable.

**At operation** in March 1972 (A.A.J.B.D.) a large cyst 8 inches in diameter in the region of the head of the pancreas was found. The common bile duct, cystic duct and gall bladder were grossly dilated. An operative cholangiogram (plate LVII) confirmed the presence of external pressure on the biliary tree. Dye entered the duodenum. A large amount of watery fluid was aspirated from the cyst. Excision was considered to be technically impossible and the cyst was drained to the outside by a T-tube.

**Plate LVII**

Operative Cholangiogram: The duodenal loop is widened and its second part is displaced laterally. The lower end of the common bile duct is very stretched and narrowed and is deviated laterally. Above this level the biliary system is dilated.

Post-operatively his jaundice settled as did his diabetes. However, on one or two occasions when his T-tube was blocked and on one occasion when it was

**Present Address:*
*Department of Surgery*
*Royal Postgraduate Medical School,*
*Hammersmith Hospital,*
*London, W.12.*

accidently pulled out, he had recurrence of his epigastric pain, diabetes and also steatorrhea. In January 1973, a year after his first operation, the cyst was drained internally (Mr. A. C. Akehurst) by anastomosing the now well formed fistula to his jejunum. He has, on one occasion since, had recurrence of his abdonimal pain when presumably the drainage of the cyst was blocked. This was accompanied by a return of the diabetes which necessitated treatment with insulin. By April 1973 his urine was free of sugar and his blood sugar levels were in the region of 90mg/100ml. When he was last seen in December 1973 he was again on insulin but did not have any abdominal complaints.

**Discussion**

Most surgeons see few pancreatic cysts of any kind, and only about one fifth of these are true cysts, the rest being pseudocysts. Solitary true cysts of the pancreas are very rare. Although no histology was done on the lining of the cyst, this was presumed to be a true cyst of the retention type (Plate LVIII).

**Plate LVIII**

Pancreatic Cystogram via T-tube: The tip of the tube is shown to lie in the pancreatic duct. The contrast medium has passed along the duct and filled a large cavity and also entered the duodenum. The second part of the duodenum is stretched around the contrast filled cavity. The contrast medium has passed retrogradely from the main pancreatic duct into the finer radicles of the glandular tissue.

Several classifications of pancreatic cysts have been proposed but that by Howard and Jordan (1960) is a very comprehensive one. (Table I).

A retention cyst is a dilatation of the pancreatic duct behind a point of obstruction. Cystic dilatation of the pancreatic ducts large enough to be of clinical importance were recognised well over 100 years ago. Virchow (1863) applied the term "Ranula pancreatica" to a marked saccular dilatation of the main pancreatic duct distal to a point of occlusion. The various causes for the occlusion are included in Table I.

**Table I**
**Classification of Pancreatic Cysts (After Howard and Jordan)**
**(Reproduced with acknowledgement)**

A. **Pseudocysts** (no epithelial lining)
1. Post-inflammatory —Inflammatory, traumatic, parasitic, secondary to neoplasm
2. Post-traumatic —E.g. Hydatid.
3. Secondary to neoplasms.
4. Secondary to parasites.
5. Idiopathic.

B. **True cysts** (have an epithelial lining)
1. Congenital:
   a. Simple.
   b. Polycystic.
   c. Fibrocystic.
   d. Dermoid.
2. Acquired:
   a. Retention. —Inflammatory, traumatic, parasitic, secondary to neoplasm
   b. Parasitic. —E.g. Hydatid.
   c. Neoplastic.
      i. Benign —Cystadenoma, angiocyst.
      ii. Malignant —Cystadenocarcinoma teratoma.

Diabetes and steatorrhea, both of which were present in this patient, are often found in patients with long standing chronic and relapsing pancreatitis, where there has been continuing destruction of pancreatic tissue (Truelove and Reynell, 1972). Georges Guillemin et al (1971) found that 16 out of 63 patients with chronic relapsing pancreatitis had diabetes mellitus. In a study of 12 cases of pancreatic cysts over a period of 18 years in Chicago only one patient needed small doses of insulin to control a slight amount of glycosuria (Lawton et al, 1954).

In this patient internal drainage was felt to be technically impossible at the first operation. The external fistula did result in loss of enzymes and also carried the risk of intermittent occlusions.

The ideal treatment of a pancreatic cyst is total excision, but often this Is not technically possible. Excision of cysts of the head of the pancreas may only be possible by pancreaticoduodenectomy. Some cysts are multilocular and hence an anastomosis to an adjoining structure may not be adequate. The cyst walls may not always be of sufficient texture to permit a safe anastomosis. Excisions also carry a higher mortality rate than drainage procedures (Warren et al, 1958).

Drainage procedures are the alternative to excision but these have the great disadvantage that a potentially malignant cystadenoma or malignant cystadenocarcinoma is not removed (Desmond et al, 1970). Drainage procedures consist of simple external drainage, marsupialization or internal drainage. Desmond et al feel that marsupialization is unsatisfactory and point out the fact that simple external drainage may lead to a persistent of prolonged fistula. External drainage, however, is simple to perform and suitable for a patient who cannot tolerate a more extensive procedure.

### Summary

A very unusual case of a solitary cyst of the pancreas is reported. The patient needed insulin for his diabetes mellitus, but as long as the cyst was draining he was free from diabetes and needed no treatment whatsoever.

### Acknowledgements

I would like to thank Mr. A. C. Akehurst for his help and encouragement and for permission to publish this report of a patient under his care.

### REFERENCES

1. DESMOND, A. M. and ROBINSON, M. R. G. (1970). Solitary cyst in the head of the pancreas. British Journal of Surgery, **57**, 209-212.
2. GUILLEMIN, G., GUILLERET, J., MICHEL, M., BERARD, P. and FEROLDI, J. (1971). Chronic relapsing pancreatitis. American Journal of Surgery, **122**, 802-807.
3. HOWARD, J. M. and JORDAN, G. L. (1960). Surgical Diseases of the Pancreas. London, Pitman Medical.
4. LAWTON, S. E., and MOSSEY, R. O. (1954) Pancreatic cysts, Archives of Surgery, **68**, 734-743.
5. TRUELOVE, S. G. and REYNELL, P. C. (1972), Diseases of the Digestive System. London, Blackwell Scientific.
6. VIRCHOW, R. (1863) Die Krankhaften Geschwulste pp. 276 (Quoted by Howard and Jordan).
7. WARREN, W.D., MARSH, W. H. and SANDUSKY, W. R. (1958). An appraisal of surgical procedures for pancreatic pseudocyst. Annals of Surgery, **147**, 903-920.

## Figures and Tables

**Plate LVII f1:**
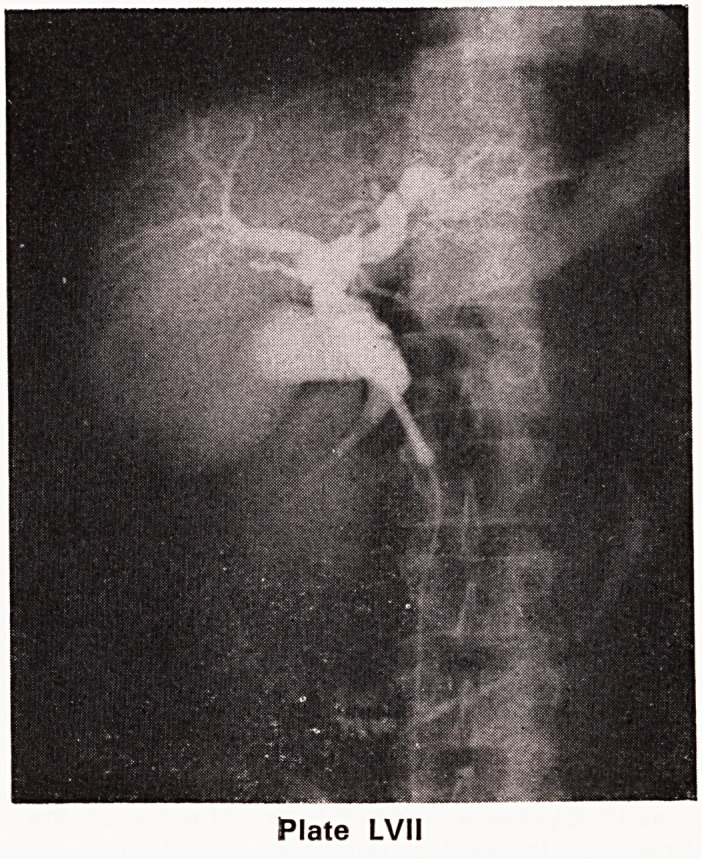


**Plate LVIII f2:**